# Quality of Life in Heart Failure Patients: The Effect of Anxiety and Depression (Patient–Caregiver) and Caregivers’ Quality of Life

**DOI:** 10.3390/jcdd12040137

**Published:** 2025-04-04

**Authors:** Athanasia Tsami, Ioannis Koutelekos, Georgia Gerogianni, Georgios Vasilopoulos, Niki Pavlatou, Antonia Kalogianni, Theodore Kapadochos, Angleliki Stamou, Maria Polikandrioti

**Affiliations:** Department of Nursing, University of West Attica, 12243 Athens, Greece; athtsami@uniwa.gr (A.T.); ikoutel@uniwa.gr (I.K.); ggerogiani@uniwa.gr (G.G.); gvasilop@uniwa.gr (G.V.); npavlatou@uniwa.gr (N.P.); antonia_cal@uniwa.gr (A.K.); kapadohos@uniwa.gr (T.K.); astamou@uniwa.gr (A.S.)

**Keywords:** anxiety, depression, quality of life, HADs, SF36, Minnesota

## Abstract

Patients with heart failure (HF) and their caregivers are a dyad inextricably linked that exert influence on patients’ quality of life (QoL). Purpose: The aim of this study was to explore factors affecting HF patients’ QoL. Factors were: (a) HF patients’ characteristics, (b) anxiety/depression of the dyad (patient–caregiver) and (c) caregivers’ QoL. Material and Methods: In this cross-sectional study, we enrolled 340 patients and 340 caregivers. Data collection was performed by the method of an interview using “The Hospital Anxiety and Depression Scale”, HADS) to assess anxiety and depression (patient–caregiver) as well as the “Minnesota Living with Heart Failure” and the “SF-36 Health Survey (SF-36)” to assess QoL (patient–caregiver, respectively). Results: From the 340 dyads who comprised the sample, 81.3% and 77.5% of patients experienced anxiety and depression, respectively, while 79.3% and 62.2% of caregivers experienced anxiety and depression, respectively. A statistically significant difference between patients and caregivers was only detected for depression (*p* = 0.001) and not for anxiety (*p* = 0.567). Patients with scores in HADS that indicate anxiety and depression had a worse QoL (total, physical, and mental). All subscales of the caregiver’s QoL were significantly associated with the patient’s QoL (*p* < 0.001) apart from the physical functioning scale. The correlation coefficients were all negative, indicating that a better caregiver’s QoL (higher SF36 scores) is associated with a better patient’s QoL (lower Minnesota scores). After controlling for the patient’s characteristics, the anxiety and depression of caregivers did not affect the patient’s QoL (confounding effect) whereas the patient’s anxiety/depression remained significant factors. Patients with anxiety and depression had 5.58 and 6.49 points, respectively, higher QoL score, meaning a worse QoL, compared to those with no anxiety/depression. Conclusions: Evaluating the impact of HF on patients’ QoL and anxiety/depression along with their caregivers permits acknowledgment of this dyadic relationship.

## 1. Introduction

Heart failure (HF) is a progressive debilitating clinical syndrome affecting not only patients but also their caregivers. According to global estimates, HF affects 26 million people, while over 960,000 new cases are diagnosed annually [1,2]. Furthermore, HF is associated with frequent hospitalizations and increased morbidity and mortality [1,2].

According to estimates, the prevalence of anxiety in HF ranges from 20% to 50% and prevalence of depression ranges from 20% to 45% [3]. The relationship between HF and anxiety and depression is bidirectional. HF may trigger anxiety and depression, whereas already established anxiety and depression exert a negative influence on HF outcomes [3]. Despite this mutual relationship, anxiety and depression are frequently under-recognized and consequently undertreated. Moreover, HF has a negative impact on patients’ quality of life (QoL), thus illustrating caregivers’ participation in care as a demanding issue [2,3,4,5].

Therefore, this dyad (patient–caregiver) is influenced by the impact of HF on their daily life, including unplanned emergency room visits, frequent decompensations, and several difficulties in self-care. All these parameters are only some of those triggering a substantial emotional burden on this dyad, such as anxiety, depression, and isolation. The shared experience is influencing their QoL [6].

Furthermore, in various cardiovascular diseases, the burden of caregivers (physical, emotional) is a crucial concept in illness trajectory [6]. Caregivers play a vital role in day-to-day HF management, and if they promptly recognize symptoms deterioration and take all measures taught by health professionals they may manage to prevent hospital readmission. This global issue acquires importance in the realms of medical care since it may reduce the financial burden on the national health system of each country. Notably, HF accounts for 1–3% of total healthcare costs, of which two-thirds emerge from hospitalization [2].

An in-depth understanding of this burden may enhance awareness among healthcare professionals and help in implementing effective and appropriate strategies to improve QoL.

The aim of this cross-sectional study was to explore factors affecting HF patients’ QoL. Factors were: (a) HF patients’ characteristics, (b) anxiety/depression of the dyad (patient–caregiver), and (c) caregivers’ quality of life (QoL).

## 2. Material-Methods

### 2.1. Design, Setting, and Period of the Study

In the present cross-sectional study, 340 hospitalized HF patients and 340 caregivers in public hospitals in Athens were enrolled from 2021 to 2024. Participants were selected using the method of convenience sampling.

In this observational cross-section, the groups of caregivers and HF patients were measured at a single point in time and the aim was to determine the relationship between the variables under study: there was no calculation of sample size [7]. As a general rule, in quantitative research studies in order to attain a safe confidence interval and an acceptable margin of error, the sample sizes start at 200 [8]. All available dyads were collected. However, during study period some recruitment issues emerged: (a) all accessible hospitalized HF patients were not accompanied by their caregivers, thus a large percentage was excluded, and (b) according to clinical observations, during the summer months (May to September) HF patients tend not to be hospitalized as their symptoms do not deteriorate.

### 2.2. Inclusion and Exclusion Criteria of the Sample

Criteria for inclusion in the study (patients and caregivers) were as follows: (i) age above 30 years; (ii) ability to write, read, and understand the Greek language; (iii) ability to read and sign the informed consent form. For patients, the exclusion criteria were as follows: (i) a history of mental illness and (ii) hospitalization for other comorbidities, apart from HF. For both patients and caregivers the exclusion criteria were as follows: (i) sight or hearing problems and (ii) receiving anxiolytics or antidepressants.

In the present study, only informal caregivers were included. These are individuals that provide regularly care to patients in the context of their relationship, such as family members. Therefore, caregivers were children or spouses (husband or wife) who were the members of family providing care to HF patients.

### 2.3. Data Collection and Procedure

Data were collected by the researcher using the method of an interview to complete the research instrument which was designed for the purposes of this study. The process of filling out the questionnaire lasted between 20 and 30 min and took place on the first day of hospital admission.

### 2.4. Research Instrument

Data were collected using the scale “The Hospital Anxiety and Depression Scale, HADS”, the “Minnesota scale”, and the SF-36 scale including patients’ and caregivers’ characteristics. In terms of patients’ characteristics, the following were recorded: gender, age, and NYHA. The following caregivers’ characteristics were recorded: gender, age, education level, employment, residency, and frequency of visits.

#### 2.4.1. Measurement of Anxiety–Depression (HADS)

For the evaluation of the mental health (depression and anxiety) of patients and their caregivers “The Hospital Anxiety and Depression Scale (HADS)” was used. This scale, which was proposed in 1983 by Zigmond AS and Snaith RP [9], consists of 14 questions that assess how patients felt during the previous week. Patients were able to answer every question on a 4-point Likert scale from 0 to 3. Seven of the fourteen questions assess depression level and the other seven anxiety level. Scores attributed to questions are summed separately for anxiety and depression. The scores assigned to questions were accumulated for the questions that assess depression and those that assess anxiety, thus leading to two scores, each with a range between 0 and 21. Higher values of scores indicate higher levels of anxiety and depression. The following classification for 2 scores has been proposed and used widely in the literature: a score of 0–7 indicates no anxiety or depression, a score of 8–10 indicates moderate levels of anxiety or depression, and a score of 11–21 indicates high levels of anxiety or depression [9,10]. In addition, in the literature the following categorization is used: a score of 0–8 indicates no anxiety or depression, and a score above 8 indicates anxiety or depression [11].

#### 2.4.2. Measurement QoL of HF Patients

The “Minnesota Living with Heart Failure” scale was used for QoL evaluation. In more detail, this scale was proposed in 1986 by Minnesota University and assesses two QoL dimensions: the physical and mental state. The scale consists of 21 items that assess the patients’ QoL within the last month. Participants answered each question on a Likert scale, with scores from 0 to 5. The scores of questions are summed separately for the physical and mental state and all scores are summed for a total QoL score. Higher scores mean a higher impact of HF on QoL, thus indicating a low QoL [12].

#### 2.4.3. Measurement QoL of HF Caregivers

For the QoL assessment in caregivers, the “SF-36 Health Survey (SF-36)” scale was used. In more detail, this scale which was created by Ware and colleagues assesses physical and mental health. The SF-36 scale consists of 36 questions exploring the following 8 dimensions: physical functioning, physical role, physical pain, general health, energy/fatigue, social functioning, emotional role, and emotional well-being. Participants answered each question on Likert-type scales. The scores of the questions are summed up separately for each of the 8 dimensions. Higher scores indicate a better QoL [13].

### 2.5. Ethical Considerations

The present study was approved by the Research Committee of two public hospitals (EΣ 900/24-92020 and EΣ 41o/27-10-2020). Participants who met the entry criteria were informed by the researcher about the purposes of the study. Written informed consent was obtained for all participants being interviewed. Data collection guaranteed anonymity and confidentiality. All subjects had been informed of their rights to refuse or discontinue their participation in the study, according to the ethical standards of the Declaration of Helsinki (1989) of the World Medical Association.

### 2.6. Statistical Analysis

Categorical data are presented with absolute and relative frequencies (%), while continuous data are presented with their mean, standard deviation, median, and interquartile range. The normality of the data was tested with the Kolmogorov–Smirnov criterion and graphically with Q-Qplots and histograms. The non-parametric Mann–Whitney test, the Kruskal–Wallis test, and the McNemar test, as well as Spearman’s rho correlation coefficient, were each used to test for the association between the patients’ QoL and their anxiety and depression along with the anxiety and depression of their caregivers. In addition, multiple linear regression was performed to estimate the effect of anxiety/depression on the patient’s QoL as well as their caregiver’s anxiety/depression and QoL. Results are presented as β regression coefficients with a 95% confidence interval (95% CI). The observed level of 5% was considered statistically significant. All statistical analyses were performed with SPSS version 28 (SPSSInc, Chicago, IL, USA).

## 3. Results

### 3.1. Sample Description (Patients)

Table 1 presents the description of the patients sample. Men accounted for the 66.5% of the patients, 85.5% were over 60 years of age, and 47.1% were of NYHA III, (New York Heart Association Functional Classification for Heart Failure). Most of the sample were pensioners (72.1%) and almost half of the sample had a primary level education (44.1%).

### 3.2. Sample Description (Caregivers)

Table 2 presents the description of the caregiver’s sample. Men accounted for 28.2% of the caregivers and 68.8% were spouses. Regarding the caregiver’s job occupation, 27.6% were private employees and 25.6% were pensioners. The majority had secondary education (38.8%) or had completed university (32.4%). Most of the caregivers who were children of the patients were 41–50 years of age (43.4%), and most of the caregivers who were spouses of the patients were 71–80 years of age (34.6%)

### 3.3. Anxiety/Depression Among the Dyad (Patients–Caregivers)

Regarding anxiety/depression of patients and their caregivers, Table 3 illustrates that 81.2% of the patients had anxiety and 77.6% depression, with a cut-off score of eight. A total of 19.1% and 14.4% of the patients had moderate levels of anxiety and depression (a score of 8–10), respectively. A total of 69.1% and 67.9% of the patients had high levels of anxiety or depression (a score of 11–21), respectively. Regarding caregivers, 79.1% had anxiety and 60.9% had depression. A total of 30.3% and 31.2% of the caregivers had moderate levels of anxiety and depression (a score of 8–10), respectively. A total of 57.1% and 39,7% of the caregivers had high levels of anxiety and depression (a score of 11–21), respectively.

A statistically significant difference between patients and caregivers was only detected for depression (*p* = 0.001) and not for anxiety (*p* = 0.500). The percentage of patients with depression was significantly higher than that of caregivers.

### 3.4. Patient’s QoL

Table 4 presents the results regarding the patient’s QoL. Patients showed moderate to poor levels of total QoL (median 75), moderate levels in mental state (median 17), and poor levels in physical state (median 36).

### 3.5. Caregiver’s QoL

Table 5 presents results regarding the caregiver’s QoL. Caregivers showed moderate to high QoL levels in the emotional role (median 66.7), moderate QoL levels in the physical role, energy/fatigue, emotional well-being, and social functionality (median 50, 50, 56, and 50, respectively). Poorer QoL levels were found in physical functioning, pain, and general health (median 19, 45, and 42, respectively).

### 3.6. Associations Between Patients’ QoL and Their Characteristics

Table 6 presents the associations between the patient’s QoL and their characteristics. The QoL total and either the physical or mental state was statistically significantly associated with gender (*p* = 0.049, *p* = 0.007, and *p* = 0.003), age (*p* = 0.049, *p* = 0.001, and *p* = 0.001), NYHA (*p* = 0.001, *p* = 0.001, and *p* = 0.001), education (*p* = 0.019, *p* = 0.002, and *p* = 0.001), and job (*p* = 0.003 and *p* = 0.001 for physical and mental state). Specifically, we found a statistically significant worse QoL for female patients, those aged over 80, those with NYHA stage IV, those with primary level of education, and unemployed patients.

### 3.7. Associations Between HADS (Patient–Caregiver) and Patient’s QoL

Table 7 presents the association between the patient’s and caregiver’s anxiety/depression and the patient’s QoL. All associations were detected to be statistically significant (all *p*-values < 0.001). Patients with scores in HADS that indicate anxiety and depression had a worse QoL in total, as well as in their physical and mental state (medians for anxiety 79, 37, and 18 vs. 57, 29, and 10; medians for depression 80, 37, and 18 vs. 57, 29, and 10). Likewise, patients whose caregivers experienced anxiety or depression had a worse QoL in total, and in their physical and mental state (medians for anxiety 77, 37, and 17 vs. 66.5, 31, and 12; medians for depression 80, 37, and 18 vs. 70, 34, and 14).

### 3.8. Association Between Patients’ QoL and Caregivers’ QoL

Table 8 presents the association between the patient’s QoL and their caregiver’s QoL. All subscales of the caregiver’s QoL were significantly associated with the patient’s QoL (*p* < 0.001) apart from the physical functioning scale. The correlation coefficients were all negative, indicating that a better caregiver’s QoL (higher SF36 scores) was associated with a better patient’s QoL (lower Minnesota scores).

### 3.9. Association Between Patient’s HADS and Caregiver’s HADS

Table 9 presents the association between the patient’s anxiety/depression and their caregiver’s anxiety/depression. All associations were statistically significant (*p* < 0.001). The percentage of patients with anxiety (HADS score > 8) was higher among those whose caregivers also had anxiety (86.5%) than those among those whose caregivers had no anxiety (60.3%). Likewise, the percentage of patients with depression (HADS score > 8) was higher among those whose caregivers had also depression (89.5%) than those whose caregivers had no depression (68.0%).

### 3.10. Effect of Anxiety and Depression (Patient–Caregiver) and Caregivers’ QoL on Patients’ QoL

Multiple linear regression was performed with dependent variables on the subscales of the patient’s QoL to estimate the effect of patient characteristics and their anxiety/depression, as well as the effect of their caregiver’s anxiety/depression on patients’ QoL (independent factors).

Table 10 shows that after controlling for the patient’s characteristics, the anxiety and depression of caregivers do not affect the patient’s QoL (confounding effect, meaning that the patient’s NYHA stage, age, and education level erase the relationship between the anxiety/depression of caregivers and the patient’s quality of life). Similarly, only one subscale from caregivers’ QoL remained statistically significant, i.e., “Energy/Fatigue” (*p* = 0.048). A one-point increase in their energy/fatigue score (better QoL) indicates a 0.18- and 0.06-points lower Minnesota score (total and mental state, respectively), which means a better patient’s QoL (b = −0.18 95% CI: −0.36–−0.01 and b = −0.06 95% CI: −0.11–−0.01, respectively).

On the other hand, the patient’s anxiety/depression remained significant factors. Patients with anxiety and depression had a 5.58 and 6.49 points, respectively, higher QoL score, meaning a worse QoL, than those with no anxiety/depression (b = 5.58 95% CI: 0.48–10.67, *p* = 0.032 and b = 6.49 95% CI: 1.41–11.58, *p* = 0.013, respectively). Moreover, patients with depression had a 2.20 points higher mental state score than those with no depression (b = 2.20 95% CI: 0.61–3.79 *p* = 0.007).

Regarding other patient characteristics, females had a 3.75 and 1.19 points higher total score of QoL and mental state than males, respectively (b = 3.75 95% CI: 0.07–7.44, *p* = 0.046 and b = 1.19 95% CI: 0.06–2.32, *p* = 0.039, respectively).

Patients with NYHA stage III and IV had a 10.82 and 16.55 points higher total score of QoL than those with NYHA stage I–II, respectively (b = 10.82 95% CI: 6.76–14.88, *p* = 0.001 and b = 16.66 95% CI: 11.15–22.16, *p* = 0.001, respectively).

## 4. Discussion

In the present study, men accounted for the 66.5% of HF patients, and 85.5% were over 60 years of age. However, in the present study, 49 patients between 30 and 60 years of age were included. It is widely known that HF is predominately a disease of the elderly. However, cardiovascular risk factors (obesity, smoking, dyslipidemia) are observed in young HF patients. Younger HF patients, with either a preserved or non-preserved ejection fraction, are frequently obese, with diabetes mellitus, and with a low prevalence of another comorbidity. However, re-hospitalization within one year after an HF event occurring for first time as well as in hospital morbidity are elevated in all groups [14,15].

Reciprocal relationships between patients and their caregivers are characterized by love and shared values or perceptions, and they exert significant influence on the health outcome in chronic illness [16]. According to estimates, 75% of HF patients encounter various daily difficulties which increase the level of dependency on caregivers [17].

Regarding anxiety/depression, 81.2% of HF patients had anxiety and 77.6% had depression, while HF patients with anxiety and depression had 5.58 and 6.49 points, respectively, higher QoL score, meaning a worse QoL, compared to those with no anxiety/depression. According to AbuRuz [18], anxiety and depression are a predictor of poor QoL in HF patients. Several mechanisms are to be held responsible for this association. In more detail, anxiety and depression stimulates the sympathetic nervous system, reduces heart rate variability, impairs platelet functioning, activates inflammatory process, and thus affects patients’ physical dimensions and consequentially their QoL.

The main sources of stress in HF include physical symptoms, complex treatment regimens, comorbidities, failure of coping mechanisms, social isolation, financial concerns, fear of death, loss of control, and frustrations with a complex healthcare system [5]. Furthermore, this comorbidity undermines the effectiveness of self-care, and along with weakness in physical and mental strength, leads to health deterioration, frequent hospitalizations, or slower recovery. According to HF management guidelines, evaluation of depression and anxiety is recommended on a systematic basis but unfortunately this is not applied in clinical practice [18,19].

In HF caregivers, anxiety and depression is not a rare phenomenon as it fluctuates from 15% to 54%, while in the present study 79.1% had anxiety and 60.9% had depression. Considering above percentages, it is of fundamental importance to explore this burden since it implies deleterious consequences either on caregiver or on the dyad (patient–caregiver). Though caregiving is essential in HF, it is associated with psychological disturbance. Interestingly, high anxiety and depression among caregivers is related with low perceived control, diminished level of functionality, and a worse relationship with the patient. In terms of patients, depression of their caregiver is related with re-hospitalizations, while high anxiety and depression are related with limited self-care [20]. Higher caregiver strain is associated with more severe patient symptoms and a low patient QoL [21], as well as with more patients ‘complaints, low satisfaction from their relationship, and limited support as perceived by patients [22]. Moreover, depressive symptoms among HF caregivers are predictive of caregiver QoL. Furthermore, functioning of family is predictive of caregiver QoL [23]. A factor that could potentially protect HF caregivers is to evaluate and enhance family dynamics since poor family functioning increases caregiver depression, which in turn increases the risk of poor caregiver QoL [23,24].

In the present study, caregivers showed a moderate QoL in physical role, energy/fatigue, emotional well-being, and social functionality, and a poorer QoL in physical functioning, pain, and general health. The role of caregivers includes management of the patient’s symptoms and dietary habits, including salt restrictions or handling with patients’ either physical or cognitive impairment. It is estimated that on a weekly basis, caregivers spend larges amount of time on the household, providing support, meeting dietary needs, and transporting patients. Re-hospitalization, implanted devices, polypharmacy, and financial worries add to this complexity. Therefore, the role of caregivers entails a physical and psychosocial burden, such as the loss of energy, poor sleep, anxiety, and social isolation. Strikingly, the fear of sudden death or acute deterioration prompt the caregiver to be in close proximity to the patient, and to the patient’s therapy management and medical visits [24,25,26]. HF caregivers frequently report unfulfilled needs, no effective communication with healthcare professionals, and a lack of alertness and response to end-of-life care [27]. In addition, HF caregivers report receiving low support (familial, organizational) for caregiving regarding financial and emotional issues [24]. All aforementioned parameters negatively affect the QoL of caregivers. Likewise, caregivers report more difficult tasks, anxiety, and a poor QoL in the physical dimension when HF patients have more severe symptoms [28]. Similarly, Hu et al. [29] demonstrated a poor QoL (physical, mental) in 251 HF patients and caregivers which was associated with daily caregiving hours, readmission within 6 months, and low social support. The same researchers showed that a poor QoL in the physical state was related to low self-efficacy.

A noticeable finding of the present study is that patients had a worse QoL (total, physical, mental) when caregivers had anxiety or depression. On the other hand, in the literature it is cited that caregivers have a worse QoL when HF patients experience depressive symptoms. In more detail, caregivers for HF patients with depression experience a high caregiving burden and a diminished QoL in comparison to those with no symptoms of depression [30]. When both members of the dyad experience depression, then a diminished QoL (mental, physical) is observed, with low communication and limited problem solving [31]. It is well known that depressive individuals have limited motivation and energy to participate in self-care. Caregivers of HF depressive patients report difficulties when trying to manage moodiness or irritability and perceive the provision of support for activities and for mobility or exercise as a toilsome task. Additionally, caregivers report difficulties arising from patients’ behavioral problems, such as memory loss, concentration, and attention. Spouses report a poor QoL when patients experience psychological disturbance, while patients have a low QoL when the spouse is experiencing high levels of stress. This concordance is explained by various mechanisms. For example, the partners of patients may experience sleep disorders, have no leisure time for their self-care behaviors, or share common lifestyle risk factors with patients (e.g., poor diet, sedentary life) [24,32]. More intriguing, Kim et al. [33] demonstrate that the physical state of a caregiver is associated with patient self-care management. As a consequence, caregivers QoL should undergo a specific diagnostic evaluation.

Given that anxiety and depression frequently keep pace with heart disease with overlapping symptoms, the alertness of health professionals to this emotional burden is a prerequisite for prompt diagnosis and treatment [34].

After controlling for the patient’s characteristics, anxiety and depression of caregivers did not affect the patient’s QoL (confounding effect, meaning that the patient’s NYHA stage, age, and education level erase the relationship between anxiety/depression of caregivers and the patient’s quality of life). Likewise, caregivers’ emotional outcomes were not associated with patients’ QoL and their behavioral and psychological symptoms in a sample of 50 dyads enrolled in acute care and followed in the community and measured over a year period (baseline, 3, 6, 12 months) [26].

The major criticality of this study that adds to the knowledge is that after controlling for the patient’s characteristics the anxiety and depression of caregivers did not affect the patient’s QoL, and that a one-point increase in energy/fatigue score (better QoL) indicated a 0.18- and 0.06-points lower Minnesota score (better patient QoL). These findings may be attributed to the role of several potential confounding factors, such as self-care, other supportive networks (relatives, children), and cognitive impairment, which are well-documented in heart failure literature.

The clinical syndrome of HF explored by the scope of the “dyad” is a crucial point for health interventions tailored to meet their needs.

### Limitations of the Study

This study has some limitations that are worthy of mentioning. Firstly, no causal relationship emerged between the variables under evaluation in the dyad (patient–caregiver) since the study was of a cross-sectional design. Secondly, the generalizability of results is limited as it used the method of convenience sampling in public hospital in Athens, which is not representative of all Greek HF patients and their caregivers. Thirdly, other confounding variables such as cognitive impairment, social support, fatigue, or self-care management were not a subject of inquiry in the present study.

The HADS, SF-36, and Minnesota scales are widely used tools that will help to compare the results with other research studies on a global scale.

## 5. Conclusions

The present study showed the following:

Patients: Moderate to poor total QoL. Caregivers: Poor QoL in physical functioning, pain, and general health.

Effect of HF patients’ characteristics on their QoL

Female patients, those aged over 80, those with NYHA stage IV, those with primary level of education, and unemployed patients all had a worse QoL.

Effect of anxiety and depression on patients’ QoL

Patients with anxiety and depression had a worse QoL (total, physical, mental). Patients whose caregivers had anxiety or depression had a worse QoL.

Effect of caregivers’ anxiety and depression on patients’ QoL

Caregivers’ anxiety and depression did not affect the patient’s QoL.

Effect of caregivers’ QoL on patients’ QoL

The “Energy/Fatigue” caregivers’ QoL subscale affected patients QoL

The present QoL, findings contribute to HF field. QoL measurement of HF patients from theoretical perspective, is important for promoting person-centered care while from clinical perspective, is important for monitoring, evaluation of changes in life during treatment and assessment of treatment effectiveness.

Nevertheless, interpretation of the results should be made with caution as these studies analyze data at one point in the time and variables under assessment may change in a next measurement or any other circumstances.

In terms of future directions, when exploring health related issues among HF patients is essential to focus on caregivers, acknowledging that this progressive clinical syndrome is a shared experience. Additionally, future orientation for research in this important area may include differential diagnosis among HF patients, treatment planning, self-care management, follow up and counselling through telephone calls or through technology.

## Figures and Tables

**Table 1:** Distribution of the sample-patients according to their characteristics (n = 340).

	n (%)		n (%)
Gender		Age (Years)	
Male	226 (66.5%)	30–40	1 (0.3%)
Female	114 (33.5%)	41–50	8 (2.4%)
NYHA		51–60	40 (11.8%)
I	14 (4.1%)	61–70	93 (27.4%)
II	97 (28.5%)	71–80	150 (44.1%)
III	160 (47.1%)	>80	48 (14.1%)
IV	69 (20.3%)		
Job		Education	
Unemployed	12 (3.5%)	Primary	150 (44.1%)
Public Servant	12 (3.5%)	Secondary	114 (33.5%)
Private Employee	23 (6.8%)	University	71 (20.9%)
Freelancer	24 (7.1%)	MSc PhD	5 (1.5%)
Household	24 (7.1%)		
Pensioner	245 (72.1%)		

**Table 2:** Distribution of the sample caregivers according to their characteristics (n = 340).

	n (%)		n (%)
Relation with Patient		Gender	
Children	106 (31.2%)	Male	96 (28.2%)
Spouses	234 (68.8%)	Female	244 (71.8%)
Job		Education	
Unemployed	8 (2.4%)	Primary	80 (23.5%)
Public Servant	31 (9.1%)	Secondary	132 (38.8%)
Private Employee	94 (27.6%)	University	110 (32.4%)
Freelancer	41 (12.1%)	MSc/PhD	18 (5.3%)
Household	78 (22.9%)		
Pensioner	87 (25.6%)		
Other	1 (0.3%)		
Age (years) for children		Age (years) for spouses	
<40	35 (33.0%)	<40	13 (5.6%)
41–50	46 (43.4%)	41–50	29 (12.4%)
51–60	20 (18.9%)	51–60	42 (17.9%)
61–70	5 (4.7%)	61–70	68 (29.1%)
71–80	0	71–80	81 (34.6%)
>80	0	>80	1 (0.4%)

**Table 3:** Description of anxiety/depression of patients and their caregivers (n = 340).

	Patients n (%)	Caregivers n (%)	Mc Nemar *p*-Value
Anxiety			0.500
No (Score ≤ 8)	64 (18.8%)	71 (20.9%)	
Yes (Score > 8)	276 (81.2%)	269 (79.1%)	
Levels of Anxiety			
Normal (0–7)	40(11.8%)	43(12.6%)	
Moderate (8–10)	65(19.1%)	103(30.3%)	
High (11–21)	235(69.1%)	194(57.1%)	
Depression			0.001
No (Score ≤ 8)	76 (22.4%)	133 (39.1%)	
Yes (Score > 8)	264 (77.6%)	207 (60.9%)	
Levels of Depression			
Normal (0–7)	60 (17.6%)	99 (29.1%)	
Moderate (8–10)	49 (14.4%)	106 (31.2%)	
High (11–21)	231 (67.9%)	135 (39.7%)	

**Table 4:** Patient’s QoL (n = 340).

	Mean (SD)	Median (IQR)
QoL Minnesota		
Total Score (Range 0–105)	71.0 (18.2)	75.0 (63.0–84.0)
Physical State (Range 0–40)	33.5 (7.6)	36.0 (31.0–38.0)
Mental State (Range 0–25)	15.5 (5.6)	17.0 (11.0–20.0)

SD: Standard deviation, IQR: interquartile range.

**Table 5:** Levels of caregiver’s QoL (n = 340).

SF36 Caregiver’s QoL	Mean (SD)	Median (IQR)
Physical Functioning (Range: 0–100)	19.7 (7.3)	19.0 (14.0–25.0)
Role physical (Range: 0–100)	56.8 (39.3)	50.0 (25.0–100.0)
Role emotional (Range: 0–100)	53.8 (36.5)	66.7 (33.3–100.0)
Energy/fatigue (Range: 0–100)	48.6 (20.0)	50.0 (30.0–65.0)
Emotional well-being (Range: 0–100)	57.6 (19.4)	56.0 (44.0–72.0)
Social functioning (Range: 0–100)	54.9 (28.2)	50.0 (37.5–75.0)
Pain (Range: 0–100)	56.1 (28.7)	45.0 (32.5–77.5)
General health (Range: 0–100)	45.0 (22.3)	42.0 (27.0–62.0)

SD: Standard deviation, IQR: interquartile range.

**Table 6:** Factors associated with patient’s QoL.

	Total Minnesota			Physical State			Mental State		
	Mean (SD)	Median (IQR)	*p*-Value	Mean (SD)	Median (IQR)	*p*-Value	Mean (SD)	Median (IQR)	*p*-Value
Gender			0.049			0.007			0.003
Male	69.4 (18.8)	74.0 (59.0–83.0)		32.7 (7.9)	36.0 (29.0–38.0)		14.8 (5.8)	16.0 (10.0–19.0)	
Female	73.9 (16.9)	77.0 (68.0–85.0)		34.8 (6.7)	37.0 (34.0–39.0)		16.8 (5.0)	18.0 (14.0–20.0)	
Age			0.049			0.001			0.001
≤60	65.9 (22.5)	70.0 (53.0–84.0)		30.6 (9.6)	35.0 (27.0–38.0)		12.9 (5.9)	13.5 (8.0–18.0)	
61–70	69.1 (18.8)	74.0 (58.0–83.0)		32.5 (7.6)	36.0 (28.0–38.0)		14.2 (6.4)	15.0 (10.0–19.5)	
71–80	72.1 (17.2)	77.0 (67.0–83.5)		34.0 (7.2)	36.5 (33.0–38.0)		16.5 (4.8)	17.5 (15.0–20.0)	
>80	77.2 (12.0)	81.0 (71.0–86.0)		36.8 (4.2)	38.0 (36.0–39.0)		17.4 (4.3)	18.0 (15.0–21.0)	
NYHA			0.001			0.001			0.001
I–II	57.6 (21.3)	61.5 (42.0–73.0)		28.1 (9.7)	30.0 (22.0–36.0)		11.7 (5.6)	12.0 (6.0–17.0)	
III	74.0 (11.8)	76.0 (67.5–82.5)		35.1 (4.8)	36.0 (34.0–38.0)		16.1 (4.6)	17.0 (14.0–20.0)	
IV	85.4 (8.6)	86.0 (81.5–91.0)		38.1 (2.7)	39.0 (38.0–40.0)		20.0 (3.5)	21.0 (19.0–22.0)	
Education			0.019			0.002			0.001
Primary	74.8 (14.9)	78.0 (70.0–85.0)		35.4 (5.6)	37.0 (34.0–39.0)		17.1 (5.0)	18.0 (15.0–21.0)	
Secondary	70.3 (18.3)	74.0 (61.0–84.0)		33.0 (7.4)	36.0 (30.0–38.0)		14.9 (5.6)	16.0 (10.0–20.0)	
University/MSc/PhD	65.9 (21.2)	71.5 (52.5–81.0)		30.9 (9.7)	35.0 (27.0–38.0)		13.5 (5.9)	15.0 (8.0–18.0)	
Job			0.119			0.003			0.001
Unemployed/Household	70.9 (25.2)	78.0 (68.0–90.0)		32.5 (10.2)	37.0 (30.0–38.5)		15.7 (6.9)	18.0 (10.0–21.0)	
Employee	66.8 (20.3)	71.0 (57.0–81.0)		30.7 (8.8)	33.0 (28.0–38.0)		12.1 (5.3)	11.0 (7.0–16.0)	
Pensioner	72.2 (16.1)	76.0 (66.0–84.0)		34.4 (6.5)	37.0 (33.0–39.0)		16.3 (5.2)	17.0 (14.0–20.0)	

**Table 7:** Association between anxiety and depression (patient–caregiver) on patient’s QoL.

	Total Minnesota			Physical State			Mental State		
	Mean (SD)	Median (IQR)	*p*-Value	Mean (SD)	Median (IQR)	*p*-Value	Mean (SD)	Median (IQR)	*p*-Value
Patients HADS Anxiety			<0.001			<0.001			<0.001
No (Score ≤ 8)	53.2 (20.3)	57.0 (35.0–69.0)		26.5 (9.9)	29.0 (18.0–35.0)		10.1 (5.5)	10.0 (5.0–14.0)	
Yes (Score > 8)	75.6 (14.5)	79.0 (70.0–85.0)		35.1 (5.8)	37.0 (34.0–39.0)		16.9 (4.7)	18.0 (14.0–20.0)	
Patients HADS Depression			<0.001			<0.001			<0.001
No (Score ≤ 8)	53.1 (19.4)	57.0 (36.0–70.0)		26.5 (9.3)	29.0 (19.0–34.0)		10.2 (5.6)	10.0 (5.0–15.0)	
Yes (Score > 8)	76.3 (13.9)	80.0 (71.0–85.5)		35.4 (5.6)	37.0 (34.0–39.0)		17.1 (4.5)	18.0 (15.0–21.0)	
Caregivers HADS Anxiety			<0.001			<0.001			<0.001
No (Score ≤ 8)	59.5 (23.6)	66.5 (35.0–78.0)		28.5 (10.3)	31.0 (21.0–37.0)		12.2 (6.5)	12.0 (6.0–17.0)	
Yes (Score > 8)	73.9 (15.3)	77.0 (68.0–85.0)		34.7 (6.2)	37.0 (33.0–39.0)		16.3 (5.0)	17.0 (13.5–20.0)	
Caregivers HADS Depression			<0.001			<0.001			<0.001
No (Score ≤ 8)	64.8 (19.6)	70.0 (55.0–80.0)		31.5 (8.7)	34.0 (28.0–38.0)		13.4 (5.9)	14.0 (9.0–19.0)	
Yes (Score > 8)	74.6 (16.7)	80.0 (69.0–86.0)		34.6 (6.7)	37.0 (33.0–39.0)		16.8 (5.1)	18.0 (14.0–20.0)	

**Table 8:** Association between patient’s QoL and caregiver’s QoL (n = 340).

	Patient’s QoL					
	Total Minnesota		Physical State		Mental State	
Caregiver’s QoL	Rho	*p*-Value	Rho	*p*-Value	Rho	*p*-Value
Physical Functioning	0.087	0.139	0.071	0.210	0.133	0.058
Role physical	−0.258	0.001	−0.243	0.001	−0.273	0.001
Role emotional	−0.295	0.001	−0.242	0.001	−0.307	0.001
Energy/fatigue	−0.365	0.001	−0.308	0.001	−0.402	0.001
Emotional well-being	−0.349	0.001	−0.293	0.001	−0.371	0.001
Social functioning	−0.342	0.001	−0.307	0.001	−0.341	0.001
Pain	−0.289	0.001	−0.266	0.001	−0.307	0.001
General health	−0.286	0.001	−0.257	0.001	−0.294	0.001

**Table 9:** Association between patient’s HADS and caregiver’s HADS (n = 340).

	Patient Anxiety			Patient Depression		
	No n (%)	Yes n (%)	*p*-Value	No n (%)	Yes n (%)	*p*-Value
Caregiver’s Anxiety			0.001			0.001
No (Score ≤ 8)	27 (39.7%)	41 (60.3%)		34 (51.5%)	32 (48.5%)	
Yes (Score > 8)	35 (13.5%)	224 (86.5%)		40 (15.4%)	220 (84.6%)	
Caregiver’s Depression			0.001			0.001
No (Score ≤8)	39 (32.0%)	83 (68.0%)		48 (40.7%)	70 (59.3%)	
Yes (Score >8)	21 (10.5%)	179 (89.5%)		24 (11.8%)	179 (88.2%)	

**Table 10:** Effect of anxiety and depression (patient–caregiver) and caregivers’ QoL on patients’ QoL (n = 340).

	Total Minnesota		Physical State		Mental State	
	b Coef (95% CI)	*p*-Value	b Coef (95% CI)	*p*-Value	b Coef (95% CI)	*p*-Value
Gender (female vs. male)	3.75 (0.07–7.44)	0.046	1.13 (−0.43–2.69)	0.157	1.19 (0.06–2.32)	0.039
Age						
≤60	ref		ref		ref	
61–70	1.44 (−3.90–6.77)	0.596	1.03 (−1.27–3.33)	0.379	0.56 (−1.09–2.21)	0.506
71–80	−1.36 (−6.98–4.27)	0.635	0.32 (−2.08–2.72)	0.793	1.50 (−0.23–3.24)	0.090
>80	1.40 (−5.63–8.43)	0.695	1.62 (−1.43–4.67)	0.296	1.56 (−0.65–3.77)	0.165
NYHA						
I–II	ref		ref		ref	
III	10.82 (6.76–14.88)	0.001	4.70 (2.98–6.43)	<0.001	2.15 (0.90–3.40)	0.001
IV	16.66 (11.15–22.16)	0.001	5.61 (3.31–7.91)	<0.001	4.35 (2.65–6.05)	<0.001
Education						
Primary	ref		ref		ref	
Secondary	−1.67 (−5.36–2.01)	0.372	−0.74 (−2.34–0.85)	0.361	−0.13 (−1.27–1.02)	0.830
University/MSc/PhD	−2.19 (−6.34–1.95)	0.299	−1.59 (−3.37–0.19)	0.080	−0.63 (−1.94–0.68)	0.343
HADS patient’s Anxiety (yes vs. no)	5.58 (0.48–10.67)	0.032	2.13 (−0.13–4.40)	0.065	1.39 (−0.23–3.01)	0.091
HADS patient’s Depress. (yes vs. no)	6.49 (1.41–11.58)	0.013	2.19 (−0.06–4.43)	0.057	2.20 (0.61–3.79)	0.007
HADS caregiver’s Anxiety (yes vs. no)	−0.75 (−5.73–4.23)	0.767	1.24 (−0.91–3.40)	0.257	−0.12 (−1.68–1.43)	0.875
HADS caregiver’s Depression (yes vs. no)	0.06 (−4.48–4.61)	0.978	−0.15 (−2.09–1.79)	0.877	0.26 (−1.14–1.66)	0.714
Caregiver’s QoL						
Physical Functioning	0.02 (−0.26–0.30)	0.886	−0.02 (−0.14–0.10)	0.787	0.03 (−0.06–0.11)	0.567
Role physical	−0.03 (−0.10–0.04)	0.414	−0.01 (−0.04–0.02)	0.452	0.00 (−0.02–0.02)	0.988
Role emotional	−0.03 (−0.10–0.04)	0.351	−0.01 (−0.04–0.02)	0.470	−0.01 (−0.03–0.02)	0.644
Energy/fatigue	−0.18 (−0.36–−0.01)	0.048	−0.02 (−0.10–0.06)	0.626	−0.06 (−0.11–−0.01)	0.048
Emotional well-being	0.07 (−0.09–0.23)	0.392	0.05 (−0.02–0.12)	0.159	0.00 (−0.05–0.05)	0.948
Social functioning	−0.01 (−0.13–0.12)	0.926	−0.02 (−0.07–0.03)	0.450	0.00 (−0.04–0.04)	0.953
Pain	0.01 (−0.12–0.13)	0.929	0.00 (−0.05–0.05)	0.910	−0.01 (−0.04–0.03)	0.652
General health	0.08 (−0.07–0.23)	0.278	0.00 (−0.06–0.06)	0.993	0.03 (−0.02–0.07)	0.214

b coef: b regression coefficient, CI: confidence interval, ref: reference category; patient’s occupation was not entered in the model due to a multicollinearity effect with the other coefficients.

## Data Availability

Data is contained within the article.
